# Induction of cervical dilation for transcervical embryo transfer in ewes

**DOI:** 10.1186/1477-7827-12-8

**Published:** 2014-01-28

**Authors:** Ivanka BR Candappa, Pawel M Bartlewski

**Affiliations:** 1 Department of Biomedical Sciences, Ontario Veterinary College, University of Guelph, Guelph ON, Canada

**Keywords:** Sheep, Ewe, Cervix, Prostaglandin E_2_, Dinoprostone, Embryo transfer

## Abstract

**Background:**

A major limitation in the application of assisted reproductive technologies in sheep arises from the inability to easily traverse the uterine cervix. The cervix of the non-pregnant ewe is a narrow and rigid structure, with 5–7 spiral folds and crypts that block its lumen. The first two folds closest to the vagina appear to be the greatest obstacle for the instrument insertion into the sheep cervix. Therefore, the dilation of the distal part of the cervix could provide the conformational change necessary to perform non-invasive transcervical procedures. The present study set out to assess the efficacy of Cervidil®, a patented dinoprostone (PgE_2_)-containing vaginal insert with a controlled-release mechanism, to safely induce sufficient cervical dilation for the purpose of transcervical embryo transfer (TCET) in cyclic ewes.

**Methods:**

The transfer of frozen-thawed ovine embryos was attempted in 22 cross-bred Rideau Arcott x Polled Dorset ewes, with or without the pre-treatment with Cervidil® for 12 or 24 h prior to TCET.

**Results:**

Cervical penetration rate was significantly improved after Cervidil® pre-treatment, with 55% (6/11) of treated versus 9% (1/11) of control animals successfully penetrated (*χ*^2^-test, p < 0.05). Within the treated ewes that were penetrated, 67% (4/6) had been exposed to Cervidil(R) for 24 h and 33% (2/6) had had a 12-h exposure (p > 0.05). Variations in the age, weight, genotype, parity, lifetime lamb production (LLP) and post-partum interval (PPI) between penetrated and non-penetrated ewes were not significant (p > 0.05). The time taken to traverse the uterine cervix was negatively correlated (p < 0.05) with the age, parity, LLP and PPI. Progesterone assays and ultrasonographic examinations performed 25 days after ET confirmed pregnancy in 2 of 7 penetrated ewes, but no fetuses were detected ultrasonographically 55 days post-TCET.

**Conclusions:**

The present results indicate a significant benefit of using Cervidil® for inducing cervical dilation during the mid-luteal phase in ewes but the reason(s) for impaired fertility after the transfer of frozen-thawed ovine embryos remains to be elucidated.

## Background

The ability to gain from the benefits of embryo transfer (ET) in commercial settings is predominantly due to the ease with which these procedures can be executed. Surgical approaches are not necessary to perform ET in most species of veterinary interest but rather the transcervical route is used. The ovine cervix, however, is a narrow and rigid structure with a complex, tortuous arrangement that precludes easy transcervical passage and intrauterine deposition of harvested embryos in recipient ewes using conventional ET catheters [1,2]. The main method used to circumnavigate this anatomical barrier involves surgical intervention; intrauterine ET in sheep is usually performed via mid-ventral laparoscopy [3,4]. Although considered moderately invasive, this procedure causes undue stress to animals, is time consuming, costly, labor intensive, and requires specialized equipment and qualified personnel. The repeated use of this technique is also a concern for animal welfare. As a result, the use of laparoscopic ET is not widespread in the sheep industry and is limited to small flocks of usually rare, expensive, heritage breeds. The cost to benefit ratio of surgical transfers is not favorable especially if the transfer to multiple recipients is required. Therefore, a reliable transcervical method of ET would be a valuable asset to the sheep breeders. Wulster-Radcliffe et al. [5] were able to achieve cervical penetration and intrauterine embryo deposition in ewes. It was found that 53.5% of embryos were retained at day 12 of pregnancy. Although promising, these results were never repeated and the development of the embryos past day 12 in utero was not assessed.

Several modified catheters have been developed to traverse cervical canal in sheep but the penetration rates remain inconsistent and unpredictable [1]. Furthermore, cervical trauma and local inflammation caused by repeated attempts to pass a rigid catheter through a highly constricted cervix may have a negative impact on the ensuing pregnancy and lambing rates [1]. A plausible solution would be to devise a means of dilating the ovine cervix prior to manipulation to enlarge the cervical lumen and thus enable the passage of ET instruments; this would greatly decrease potential cervical injury and its associated detrimental effects. Cervical dilation could also increase the ease of transcervical ET making it less dependent on operators’ skills and decreasing the amount of time taken to perform the procedure. Dilation of merely the first two caudal folds of the cervix, which are the most constricted and misaligned, would be sufficient to significantly facilitate transcervical instrument passage [1].

An ideal pharmacological agent to induce cervical dilation for the purpose of ET in ewes must be designed for easy delivery and cause minimal discomfort to the animal [1]. Such an agent should not yield adverse residual effects, neither maternal nor embryonic. In particular, it should not induce uterine atony or hyperstimulation as both of these conditions could interfere with the ability of transferred embryos to successfully implant in the endometrial layer. Cervidil® is a vaginal insert containing synthetic analog of prostaglandin E_2_ (PgE_2_). It has been established as a safe agent to induce cervical dilation in periparturient women. Cervidil® releases PgE_2_ at a constant rate to sustain physiological concentrations of the prostaglandin, similar to those observed at early stages of labor, and promoting cervical ripening without strong concomitant effects on uterine contractility. We proposed that the application of Cervidil® to cyclic sheep may facilitate transcervical embryo deposition.

Hence, the present study was undertaken to assess the utility of Cervidil® as an inducer of cervical dilation for transcervical ET in ewes. The emphasis was on the dilatory effects of Cervidil® inserts and so the embryos from only one source (frozen-thawed ovine embryos) were used and no comparisons between fresh or frozen/*in vitro*- or *in vivo*-produced embryos were attempted in the present trial.

## Methods

### Experimental animals

The present experiment utilized clinically healthy, sexually mature Rideau Arcott x Polled Dorset ewes with no previous transcervical or laparoscopic artificial insemination or ET attempts, superovulatory treatments or reproductive tract surgeries. Animals were housed in the Ponsonby Sheep Research Station near Fergus, ON, Canada (latitude: 43°42′N, longitude: 80°22′W). The ewes were fed a maintenance diet of hay, and had ad libitum access to fresh water and cobalt-iodized salt licks. Preliminary statistical analyses revealed no significant differences in the mean age, weight, breed, parity, lifetime lamb production and post-partum interval across all treatment and control groups of ewes in this study (Table 1). All experimental procedures executed were in compliance with the guidelines of the Canadian Council on Animal Care and had been approved by the local Animal Care Committee in Guelph, ON, Canada.

**Table 1:** The ewe-related characteristics for all treatment and control groups of cyclic Rideau Arcott x Polled Dorset ewes used in the transcervical embryo transfer (TCET) trial

**Variable/*Group***	***Treatment***		***Control***	
	***12-h *** **(n = 5)**	***24-h *** **(n = 6)**	***12-h *** **(n = 6)**	***24-h *** **(n = 5)**
**Age** (days)	1392 ± 171 (812 to 2121)	1686 ± 132 (1307 to 2066)	1293 ± 176 (958 to 2067)	1340 ± 65 (1241 to 1573)
**Weight** (kg)	79.0 ± 3.1 (71 to 80)	81.6 ± 3.3 (71 to 90)	78.4 ± 6.7 (52 to 102)	82.2 ± 4.2 (70 to 95)
**Breed** (% Rideau Arcott)	75.0 ± 7.2 (62.5 to 100)	50.0 ± 6.2 (31.3 to 68.8)	58.3 ± 8.5 (25 to 81.3)	62.5 ± 5.6 (43 to 75)
**Parity**	2.2 ± 0.5 (1 to 4)	3.0 ± 0.4 (2 to 4)	1.8 ± 0.5 (1 to 4)	1.6 ± 0.4 (1 to 3)
**Lifetime lamb production** (lambs/ewe)	4.7 ± 1.1 (2 to 9)	6.2 ± 1.0 (3 to 10)	3.7 ± 1.3 (1 to 9)	3.2 ± 1.1 (1 to 7)
**Post-partum interval** (days)	226 ± 1 (226 to 229)	295 ± 36 (227 to 451)	316 ± 45 (225 to 454)	399 ± 151 (226 to 1001)

### Superovulation and embryo recovery/freezing

In December, a total of 16 ewes were superovulated using a standard superovulatory protocol [6]. Prior to embryo collections, each ewe was anaesthetized with 0.2 mg/kg b.w. of xylazine i.m. (Rompun®; Bayer Animal Health, Etobicoke, ON, Canada) followed by 5 mg/kg b.w. of ketamine (Vetalar®; Bioniche Animal Health Canada Inc., Belleville, ON, Canada), administered i.v. ~10 min later. The uterine horns were exteriorized through a ventral midline incision 10 cm cranial to the udder. A stab incision at the base of the uterine horn close to the uterine bifurcation allowed for the insertion of a Foley catheter, which was then inflated with air until the tissue in contact with the ballooned area was taut. An open-ended 1/5 14-cm tom-cat catheter was introduced through a small puncture made at the utero-tubal junction in order to inject 50 ml of flushing medium (phosphate buffered saline + 1% bovine serum albumin + penicillin + streptomycin) into the uterine horn. This fluid was forced through the small opening in the Foley catheter at the base of the uterine horn and then collected into a sterile container; the process was repeated for the second uterine horn. An experienced technician immediately recovered from the collected fluid all embryos that had developed to the blastocyst or morula stage, using a stereomicroscope at 40X image magnifications. The morphological quality of the embryos was assessed according to the embryo evaluation system originally developed by Lindner and Wright for the bovine species [7]; embryos were classified as excellent, good, fair or poor (Grades 1–4). Following the evaluation, a total of 84 transferable quality embryos (excellent to fair), including 56 blastocysts and 28 morulae, were placed in Dulbecco-modified phosphate buffered saline (DMPBS)-based freezing medium solution containing 10% cell culture grade glycerol, 0.4% bovine serum albumin and 0.1 M sucrose (ViGro® Freeze Plus; Bioniche Animal Health Canada Inc., Belleville, ON, Canada). The embryos were grouped in pairs or triplets of blastocysts/morulae, packaged with media into 0.25-cc straws, and subsequently frozen using the FTS Systems Bio-Cool™ Controlled Rate Freezer (FTS Systems, Stone Ridge, NY, USA). The freezing protocol was as follows: starting temperature: −7°C for 10 min; freezing rate: 0.3°C/min; holding temperature: −35°C for 10 min; embryos plunged into liquid nitrogen.

### Cervidil®

**Figure 1:**
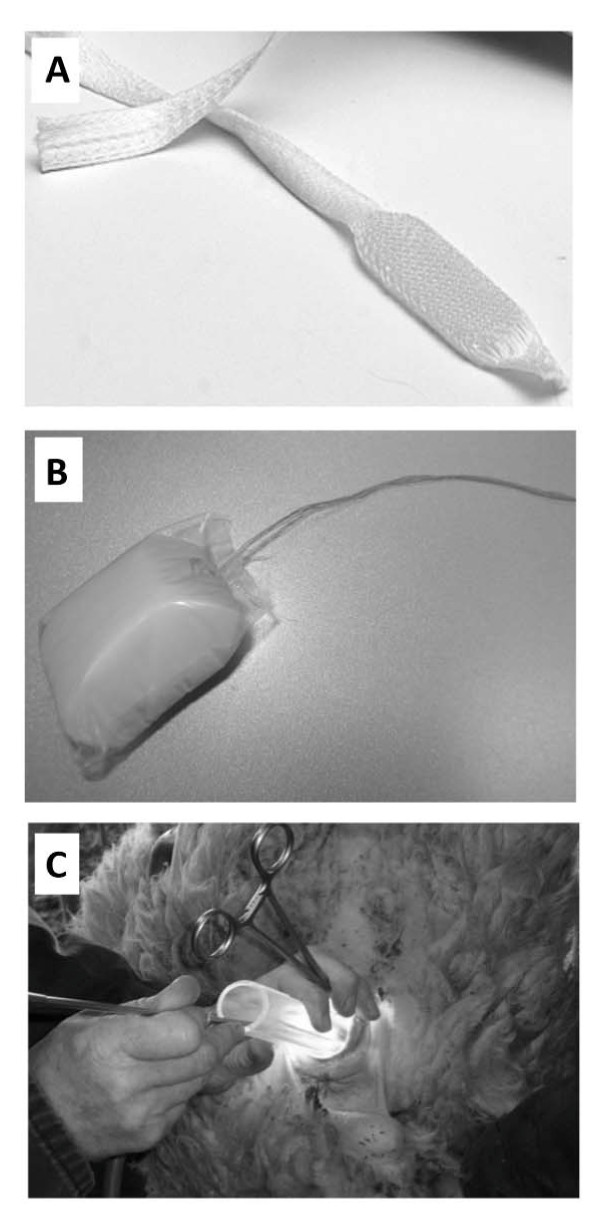
**Materials and tools used during Cervidil® treatment and transcervical embryo transfer (TCET) in ewes. (A)** Cervidil®^®^ vaginal insert; **(B)** Manufactured vaginal plugs used to prevent premature loss of Cervidil® inserts from the vaginal canal in ewes; **(C)** An illuminated vaginal canal of the ewe with a plexiglass vaginoscope inserted. Forceps are used to retract the tissue surrounding the cervical os and the tip of a modified insemination gun is inserted into the cervical opening.

The active ingredient in Cervidil® vaginal inserts (Ferring Pharmaceuticals Inc., North York, ON, Canada) is a synthetic analogue of prostaglandin E_2_ (PgE_2_), pharmacologically known as dinoprostone and chemically as 11α-15S-dihydroxy-9-oxo-prosta-5Z,13E-dien-1-oic acid (molecular formula: C_20_H_32_O_5_). It consists of a flat, thin, semi-opaque, rectangular-shaped pessary measuring 29 mm × 9.5 mm and 0.8 mm in thickness, contained within a slim, polyester knitted pouch with an attached wide cord allowing convenient retrieval of the product from the vaginal canal (Figure 1A). The pessary is comprised of 241 mg of a hexanetriol/macrogol 8000/isocyanate cross-linked hydrogel copolymer matrix with 10 mg of dinoprostone dispersed within it. This specific configuration allows for the slow release of a relatively constant dose of ~0.3 mg of dinoprostone per hour. Once the insert is placed in a moist environment, it will absorb water, swell slightly and begin releasing dinoprostone. All the components of the insert are non-toxic and have been well tolerated in placebo-controlled trials [8-10] and clinical settings [11].

Considering that the Cervidil® insert is designed for use in women remaining in a recumbent supine position, the administration of the insert had to be modified to better suit its use in ewes that are ambulatory during the entire treatment period. Small rectangular blocks of hypoallergenic spongy foam measuring 5 cm × 2.5 cm × 2.5 cm were each attached to a single, flat 12-cm long knitted fabric cord, and the sponge portion was sealed with a close-fitting, thin layer of non-toxic, malleable, transparent polyethylene resin (Figure 1B) to prevent the absorption of dinoprostone by the sponge. These manufactured “plugs” were used to prevent the Cervidil® inserts from being lost prematurely by positioning the sponges caudally to the Cervidil® insert inside the vagina, limiting the movement of the Cervidil® insert within the vaginal canal. The sponges were also used as placebo inserts, without Cervidil®, in the subsets of designated control ewes.

### Experimental procedures

#### Embryo transfer

Twenty-two ewes were fitted with controlled internal drug releasing (CIDR) devices (EAZI-BREED™ CIDR® Sheep Insert; Pfizer Animal Health, Mt. Eden, Auckland, New Zealand) on designated day 0 during the breeding season (February). On day 12, CIDRs were removed and 500 IU of PMSG (Folligon®; Intervet Canada Ltd., Whitby, ON, Canada) were administered intramuscularly. On the morning of day 19, food and water were withdrawn in preparation for transcervical embryo transfer (TCET). At this time, eleven ewes were randomly allocated to the 24-h Cervidil® treatment group and respective controls. The Cervidil® inserts were placed in the vagina using a plastic Veramix® sponge applicator, which was cleaned and disinfected before each use. For the designated treatment ewes, a Cervidil® insert was placed across the front end of a placebo sponge with both the attached cords from the insert and the sponge lying in the same direction. Both units were then pushed half-way into the barrel of the applicator with the plunger. The barrel was then gently inserted into the vagina with the cords pointing caudally. The plunger was used to push both the sponge and the Cervidil® insert out of the end of the barrel securing the insert in the proximity of the cervix and the applicator was removed. The cord of the Cervidil® insert and the cord of the sponge were tied together and remained visible outside of the vulva. Control ewes were fitted with a placebo sponge without a Cervidil® insert in the same fashion as described above. The remaining 11 animals were allocated to the 12-h Cervidil® exposure group and respective controls.

TCET was performed by a veterinarian experienced in the Guelph system transcervical artificial insemination who remained unaware of which ewes had been assigned to each group. Straws containing the embryos were thawed in a 38°C water bath. Once thawed, embryo quality was assessed via microscopy by an experienced technician and those that were of acceptable quality post-thawing were transferred to a culture dish containing a commercially available holding media (ViGro® Holding Plus, Bioniche Animal Health, Belleville, ON, Canada). The embryos were packaged with holding media into 0.5-cc semen straws and kept in an incubator at 38°C. Only Grade 1 embryos were used for transfer in the present trial. TCET was performed on the ewes in the same sequence as that used for the CIDR removal and the insertion of Cervidil® inserts and sponge plugs such that consistency in the time from Cervidil® insertion to the start of TCET for the groups of the 12- and 24-h exposure designations was maintained. A single straw containing 3 blastocysts was loaded into the modified Cassou insemination gun used for the Guelph system TCAI [1] and ewes were immobilized in the Polvendale Commodore hoof trimming cradle (Polvendale Equipment, London, England). Each ewe was positioned and secured in a dorsal recumbency with the hindquarters elevated at 30° to 40°; this positioning has been shown to produce the least amount of stress and also aids in elongating the cervix, hence facilitating cervical penetration. The Cervidil® insert and/or the sponge were removed, a plexiglass vaginoscope (tubular speculum) covered with water-soluble lubricant was inserted into the vagina, and a penlight was used to visualize the cervical os and associated vaginal folds. The tissue surrounding the cervical os was grasped with 26-cm Bozeman forceps and then gently retracted approximately 5 to 10 cm using the forceps until taut (Figure 1C). Once the os was clearly visible, the pre-loaded insemination gun was introduced into the vagina through the speculum and the tip of the pipette was placed in the os. The gun was then rotated slightly, advancing the pipette carefully through each cervical ring. It became evident that the entire cervix had been traversed and the pipette tip had entered the body of the uterus when resistance to the gentle pressure required to advance the pipette forward was no longer felt. The plunger of the gun was then depressed expelling the entire content of a straw directly into the uterus close to the bifurcation. Cervical manipulation with the insemination pipette was only attempted for a maximum of 3 min because if complete penetration was not achieved at this point in time, any further cervical manipulation may have led to injury. Embryos were not expelled if the pipette tip could not be advanced into the uterus in order to avoid pathologic pregnancy. Immediately after this procedure, a straw and a tip of the insemination gun were thoroughly rinsed with the holding media and the fluid was examined under the microscope to ascertain that all embryos were deposited in the uterus.

#### Blood sampling and hormone assays

Jugular blood samples were drawn on days 19, 20 (24 and 12 h before and just prior to TCET), 29 and 30. Blood collected from all ewes into 10-ml Becton Dickinson red-top vacutainers (Franklin Lakes, NJ, USA) was kept at room temperature for approximately 24 h in order to promote clotting prior to centrifugation at 1,500 × *g* for 10 min. Serum was then harvested and stored at −20°C for radioimmunoassay (RIA) analysis at a later date. All samples were analyzed for concentrations of estradiol 17-β (E_2_) and progesterone (P_4_) by validated RIAs [12]. The sensitivities of assays were 1.0 pg/ml and 0.03 ng/ml, for E_2_ and P_4_ assays, respectively. The range of standards was from 1.0 to 50 pg/ml and from 10 pg/ml to 10 ng/ml, in the E_2_ and P_4_ RIAs, respectively. The intra-assay coefficients of variation (CVs) for reference sera with mean E_2_ concentrations of 5.6 or 11.5 pg/ml were 7.1% and 13.7%, respectively. The intra-assay CVs for reference sera with mean P_4_ concentrations of 1.32, 6.44 or 9.54 ng/ml were 8.4%, 2.9% and 0.8%, respectively.

#### Ultrasonographic pregnancy detection

Twenty-five days post-TCET, all ewes were subjected to transrectal ultrasonographic imaging with an Aloka 900 SSD portable ultrasound system (Hitachi Aloka Medical Ltd., Tokyo, Japan) and a rigid 7.5-MHz transrectal transducer. Pregnancy detection was repeated at 55 days post-TCET using a 3.5-MHz transabdominal transducer connected to the B-mode ultrasound scanner (HS-2000; Honda Electronics Ltd., Toyohashi, Japan).

#### Data analyses

The statistical tools used to analyze the results included *χ*^2^ tests (Brandt and Snedecor formula) for the analysis of proportions; Student *t*-test and one-way analysis of variance (ANOVA) for comparison of single-time point variables between two or multiple groups, respectively; two-way ANOVA (general linear model) for assessment of repeated measurements and Pearson Product Moment correlations; all using the SigmaStat® statistical analysis software package (Systat Software Inc.; Chicago, IL, USA). Ewe-related parameters that were considered of importance for this study were: the age on the day of TCET, expressed in number of days; the body weight at the start of the study period; the breed, defined as the percentage of Rideau Arcott in the crossbred Rideau Arcott x Polled Dorset genotype; the total number of pregnancies over the lifetime prior to start of the study period (i.e., ewe parity); the total number of lambs produced and delivered vaginally over the lifetime (referred to as lifetime lamb production), and the time elapsed between the last lambing date and the date of TCET (the post-partum interval).

The “penetrated ewes” referred to successful embryo transfers involving complete cervical penetration and intrauterine embryo deposition while “non-penetrated ewes” described unsuccessful transfers consisting of incomplete or a lack of cervical penetration with no embryo deposition. Three main variables were assessed: i. the “cervical penetrability”, defined as the proportion/percentage of ewes in which the entire cervix could be traversed using the inseminating pipette and culminating in uterine embryo deposition; ii. the total time taken, in seconds, to complete the procedure, from the initial insertion of the pipette to the deposition of embryos (penetrated ewes) or pipette withdrawal (non-penetrated ewes), referred to hereafter as “TT”; and iii. the time of the final attempt, in seconds, or the time from the final insertion of the pipette to embryo deposition in cases where repeated repositioning of the pipette within the reproductive tract of a ewe was necessary (referred to as “FT”). Statistical significance was defined as p < 0.05 and all results were expressed as mean ± SEM.

## Results

In terms of overall cervical penetrability, 32% (7/22) of ewes were successfully penetrated. Of those that were penetrated, 86% (6/7) had been treated with Cervidil® and 14% (1/7) was an untreated control. Within non-penetrated ewes, only 33% (5/15) had been treated whereas 67% (10/15) had not received treatment. Of the non-penetrated ewes primed with Cervidil®, 60% (3/5) had a 12-h Cervidil® treatment and 40% (2/5) a 24-h exposure. Within the treated ewes that had been penetrated, 67% (4/6) had been exposed to Cervidil® for 24 h and the remaining ewes had had a 12-h exposure. When comparing the treatment and control groups overall, the proportion of successfully penetrated ewes (55%, 6/11) was significantly greater in the Cervidil®-treated animals compared with controls (9%, 1/11). Within this subset of ewes, the 24-h treatment group exhibited significantly greater cervical penetration rate than respective controls, but the difference between 12-h treatment group and corresponding controls was not significant (Table 2). Serum E2 concentrations and E_2_ : P_4_ ratios declined (p < 0.05) from 24 h before to the time of TCET in both the 12-h Cervidil®-treated ewes and their respective controls (Table 2). A significant rise in circulating P_4_ concentrations occurred over the 24 h before TCET in the 24-h treated ewes and between 24 and 12 h prior to TCET in the 12-h control group (Table 2).

**Table 2:** **The cervical penetrability, total time taken to perform the procedure (TT), time of the final attempt (FT) and estradiol/progesterone (E**_**2**_ : P_**4**_) **concentrations/ratios for the Cervidil®-treated cyclic Rideau Arcott x Polled Dorset ewes and their respective controls**

**Variable/*Group***	***Treatment***		***Control***	
	***12-h *** **(n = 5)**	***24-h *** **(n = 6)**	***12-h *** **(n = 6)**	***24-h *** **(n = 5)**
**Cervical penetrability**	2/5 (40%)	4/6 (67%)*	1/6 (16%)	0/5 (0%)*
**TT** (sec)	168 ± 22	110 ± 29	170 ± 28	180 ± 26
**FT** (sec)	38 ± 23	27 ± 5	154	-
**E**_**2 **_**– 24 h prior** (pg/ml)	2.4 ± 0.4†	1.8 ± 0.4	2.9 ± 0.4†	1.5 ± 0.2
**E**_**2 **_**– 12 h prior** (pg/ml)	1.9 ± 0.3	1.7 ± 0.4	2.6 ± 0.5	1.6 ± 0.5
**E**_**2 **_**– TCET** (pg/ml)	1.7 ± 0.3†	1.7 ± 0.4	1.9 ± 0.5†	1.2 ± 0.2
**P**_**4 **_**– 24 h prior** (ng/ml)	3.4 ± 0.6	2.6 ± 0.4§	2.7 ± 0.3§‡	2.9 ± 0.6
**P**_**4 **_**– 12 h prior** (ng/ml)	3.8 ± 0.4	4.0 ± 0.7	3.5 ± 0.5§	3.4 ± 0.6
**P**_**4 **_**– TCET** (ng/ml)	4.1 ± 0.5	3.8 ± 0.6§	3.7 ± 0.6‡	3.6 ± 0.4
**E**_**2 **_**: P**_**4 **_**– 24 h prior**	0.9 ± 0.3‡	0.8 ± 0.3	0.9 ± 0.2•	0.6 ± 0.1
**E**_**2 **_**: P**_**4 **_**– 12 h prior**	0.5 ± 0.1	0.5 ± 0.07	0.8 ± 0.3	0.6 ± 0.3
**E**_**2 **_**: P**_**4 **_**– TCET**	0.4 ± 0.08‡	0.5 ± 0.2	0.6 ± 0.2•	0.4 ± 0.07

Due to low numbers of penetrated control ewes, there are no comparisons of FT between the treatment and control groups.

A significant difference (p < 0.05) exists between the two values denoted by the same symbols: *within rows; †§‡• within columns.

The variation in the age, weight, breed, parity, lifetime lamb production and post-partum interval among the penetrated and non-penetrated ewes in this experiment was not significant (p > 0.05, Table 3). Similarly, serum P_4_ and E_2_ concentrations and E_2_ : P_4_ ratios at 24 and 12 h prior to TCET and at the time of TCET did not vary between penetrated and non-penetrated ewes (p > 0.05, Table 3). Serum E_2_ concentrations and E_2_ : P_4_ ratio declined (p < 0.05) from 24 h before to just prior to TCET, whereas P_4_ levels increased from 24 to 12 h before TCET in non-penetrated ewes (Table 3). In the successfully penetrated ewes, serum concentrations of P4 increased and values for E_2_ : P_4_ ratio declined from 24 h before to the time of TCET (p < 0.05, Table 3).

**Table 3:** A comparison of ewe-related parameters, and estradiol/progesterone concentrations/ratios (at 24 h prior to transcervical embryo transfer (TCET) and at the time of TCET) between the penetrated and non-penetrated Rideau Arcott x Polled Dorset ewes

**Variable/*Group***	***Penetrated *** **(n = 7)**				***Non-penetrated *** **(n = 15)**			
	***Treatment *** **(n = 6)**		***Control *** **(n = 1)**		***Treatment *** **(n = 5)**		***Control *** **(n = 10)**	
	***12-h *** **(n = 2)**	***24-h *** **(n = 4)**	***12-h *** **(n = 1)**	***24-h *** **(n = 0)**	***12-h *** **(n = 3)**	***24-h *** **(n = 2)**	***12-h *** **(n = 5)**	***24-h *** **(n = 5)**
**Age** (days)	1524 ± 155				1391 ± 88			
**Weight** (kg)	80.6 ± 3.1				80.0 ± 2.9			
**Breed** (% Rideau Arcott)	58.1 ± 8.7				63.8 ± 4.1			
**Parity**	2.3 ± 0.5				2.1 ± 0.3			
**Lifetime lamb production**	5.0 ± 1.2				4.2 ± 0.7			
**Post-partum interval** (d)	261 ± 20				354 ± 70			
**E**_**2 **_**– 24 h prior** (pg/ml)	2.0 ± 0.4				2.0 ± 0.2†			
**E**_**2 **_**– 12 h prior** (pg/ml)	1.6 ± 0.2				2.1 ± 0.3			
**E**_**2 **_**– TCET** (pg/ml)	1.6 ± 0.3				1.7 ± 0.2†			
**P**_**4 **_**– 24 h prior** (ng/ml)	2.8 ± 0.4§				3.0 ± 0.3§‡			
**P**_**4 **_**– 12 h prior** (ng/ml)	3.3 ± 0.4				3.9 ± 0.3§			
**P**_**4 **_**– TCET** (ng/ml)	3.8 ± 0.5§				3.8 ± 0.3‡			
**E**_**2 **_**: P**_**4 **_**– 24 h prior**	0.9 ± 0.3†				0.8 ± 0.1•			
**E**_**2 **_**: P**_**4 **_**– 12 h prior**	0.6 ± 0.1				0.6 ± 0.1			
**E**_**2 **_**: P**_**4 **_**– TCET**	0.5 ± 0.09†				0.5 ± 0.1•			

Within columns, a significant difference (p < 0.05) exists between the two values denoted by the same symbols: †§‡•.

The total time (TT) required to perform the procedure and recorded in all ewes or in the penetrated animals only was not correlated with the ewe-related and endocrine parameters or with the VMI values determined in the present experiment (p > 0.05, Table 4). However, the duration of the final attempt (FT) preceding successful cervical penetration and uterine deposition of embryos was negatively correlated (p ≤ 0.05) with the ewes’ age, parity, lifetime lamb productivity and the duration of the post-partum interval (Table 4).

**Table 4:** Summary of correlations between the ewe-related characteristics and estradiol/progesterone concentrations/ratios at 24 h prior to transcervical embryo transfer (TCET) and at the time of TCET with the total time taken to perform the procedure (TT) and the time of the final attempt (FT) (the latter for penetrated ewes only) determined in 22 Rideau Arcott x Polled Dorset ewes

**Variable/*Outcomes***	***Total time *** **(sec)**	***Total time *** **(sec)**	***Time of the last attempt *** **(sec)**
	**(All ewes, n = 22)**	**(Penetrated ewes only, n = 7)**	**(Penetrated ewes only, n = 7)**
**Age** (days)	r = −0.26, p = 0.25	r = −0.21, p = 0.29	**r = −0.59, p < 0.05**
**Weight** (kg)	r = 0.0007, p = 0.98	r = 0.01, p = 0.96	r = 0.02, p = 0.95
**Breed** (% Rideau Arcott)	r = −0.16, p = 0.49	r = −0.02, p = 0.93	r = −0.11, p = 0.71
**Parity**	r = −0.12, p = 0.56	r = −0.12, p = 0.56	*r = −0.52, p = 0.05*
**Lifetime lamb production**	r = −0.07, p = 0.76	r = 0.05, p = 0.81	**r = −0.59, p < 0.05**
**Post-partum interval** (days)	r = −0.27, p = 0.23	*r = −0.35, p = 0.08*	**r = −0.60, p < 0.05**
**E**_**2 **_**– 24 h prior** (pg/ml)	r = −0.31, p = 0.18	r = −0.22, p = 0.29	r = −0.31, p = 0.28
**E**_**2 **_**– 12 h prior** (pg/ml)	r = −0.03, p = 0.90	r = −0.07, p = 0.72	r = −0.36, p = 0.20
**E**_**2 **_**– TCET** (pg/ml)	r = 0.08, p = 0.72	r = 0.02, p = 0.93	r = −0.16, p = 0.59
**P**_**4 **_**– 24 h prior** (ng/ml)	r = −0.16, p = 0.51	r = −0.22, p = 0.30	r = 0.19, p = 0.51
**P**_**4 **_**– 12 h prior** (ng/ml)	r = −0.15, p = 0.51	r = −0.15, p = 0.45	r = 0.44, p = 0.11
**P**_**4 **_**– TCET** (ng/ml)	r = −0.31, p = 0.16	*r =* −*0.34, p = 0.08*	r = 0.22, p = 0.45
**E**_**2 **_**: P**_**4 **_**– 24 h prior**	r = −0.12, p = 0.61	r = −0.04, p = 0.84	r = −0.39, p = 0.16
**E**_**2 **_**: P**_**4 **_**– 12 h prior**	r = 0.06, p = 0.79	r = 0.04, p = 0.84	*r = −0.52, p = 0.06*
**E**_**2 **_**: P**_**4 **_**– TCET**	r = 0.22, p = 0.31	r = 0.21, p = 0.29	r = −0.40, p = 0.15

Significant correlations are indicated by the bold font and correlations approaching to significance (p < 0.10) are italicized. r - coefficient of correlation.

Serum P_4_ concentrations on days 29 and 30 of the experiment (9 and 10 days post-TCET, respectively) declined to basal levels in all but two animals. Ultrasonographic pregnancy detection performed 25 days post-TCET confirmed that the two ewes (one from the 12-h and one from the 24-h treatment group) were pregnant; however, a subsequent ultrasonographic scan on day 55 post-TCET revealed that these pregnancies had not been sustained.

## Discussion

The extremely low proportion of control ewes successfully penetrated during the present TCET trial indicates that the ovine cervix is highly constricted in the luteal phase of the estrous cycle. The application of Cervidil® has been met with a sizeable dilation of the cervix, especially after the 24-h exposure. The average total time taken to penetrate the cervix was 1–2 min and this is within the range of TT observed in Cervidil®-treated ewes from the transcervical artificial insemination (TCAI) trials in the breeding (unpublished results) and non-breeding seasons [13].

Kershaw-Young et al. [14] have shown that PgE_2_ mediates its effects on the cervix through four receptors, EP_1_ through EP_4_; EP_2_ and EP_4_ especially have been found to be involved in cervical dilation. EP_2_ and EP_4_ mRNA is expressed in the sheep cervix throughout the estrous cycle. In fact, Kershaw-Young et al. [14] revealed that a gradient of EP_2_ and EP_4_ mRNA expression existed along the cervix such that the greatest expression was found in the vaginal and mid-cervical region and the lowest in the uterine region. This indicates that the vaginal region may actually be more responsive to the effects of PgE_2_, and considering that dilation of merely the first two caudal folds is thought to produce sufficient cervical dilation for the purpose of TCAI/ET, the local application of Cervidil® may be beneficial. Although the EP_2_ mRNA has been shown to be highest prior to the LH surge, the EP_4_ mRNA expression remains constant over the estrous cycle. The direct binding of locally applied PgE_2_ to constitutively expressed receptors may lead to PgE_2_-induced cervical dilation during diestrus.

Cervical dilation prior to parturition is initially induced by a decline in progesterone with a concomitant increase in estradiol [1]. However, the notion that steroid hormone milieu may influence the effect of Cervidil® on the dilation of ovine cervix in diestrous ewes was not supported in this study. Serum concentrations of E_2_, P_4_ and E_2_ : P_4_ ratios generally did not vary between penetrated and non-penetrated ewes, and negative correlations between TT and P_4_ levels at TCET and between FT and E_2_ : P_4_ ratios at 12 h before TCET in successfully penetrated ewes only approached to significance.

Windsor et al. [15] found a significant effect of ewe parity on cervical penetration rates in synchronized ewes during seasonal anestrus but not in normally cycling, non-synchronized ewes in the breeding season. Some studies have reported a correlation between the breed and cervical penetrability [16], which may be due to the morphological differences in cervical structure between various genotypes of ewes; in general, the breeds with higher cervical complexity exhibited a lower degree of cervical penetration. There was an apparent effect of breed (specifically, the % of Rideau Arcott genotype) on the penetration rates in the present diestrous ewes. Kaabi et al. [16] also described a significant correlation of ewe age with cervical penetrability in a group of ewes within a similar age range to that in the present study; however, this may be confounded by factors such as parity. Significant negative correlations between FT and the ewes’ age, parity and lifetime lamb production were found in this study. Buckrell et al. [17] suggested that penetration success in the ewes undergoing TCAI was affected by the post-partum interval; a shorter interval was shown to significantly increase penetration rate. However, Windsor et al. [15] reported that cervical penetration rate was not affected by an increase in post-partum interval at TCAI, from 12 to 26 wks after lambing. The results from the present study have indicated that the duration of the post-partum interval from 225 to 1001 days (32 to 143 wks) was inversely related to the ease of cervical penetration or the time taken to traverse the cervix and deposit embryos.

The ultimate testament to the success of Cervidil®-facilitated TCET would be corroboration with sound fertility data comparable to laparoscopic ET or natural mating. In the ewes of the present study, serum P_4_ measurements and ultrasonographic imaging enabled the detection of two pregnancies, which were not subsequently maintained until day 55 post-TCET. There are many factors that may have contributed to the low fertility observed in this experiment. The transfer of frozen-thawed ovine embryos is usually associated with lower pregnancy rates compared with the transfer of freshly obtained embryos [3]. An additional factor that may have contributed to low fertility could have been embryo handling. Considering that the straw containing embryos is loaded into the insemination gun before cervical penetration is attempted, there is a considerable amount of time in which the embryos are exposed to external environment before they are deposited. Minimizing this exposure time would be beneficial since embryonic cells can be very sensitive to even minor fluctuations in temperature or air pressure or to possible contact with pathogens.

Sayre and Lewis [18], and Campbell et al. [19] have suggested that the transcervical method itself may have detrimental effects on fertility. Mechanical stimulation during manipulation of the cervix may damage the epithelial lining and result in the activation of sensitive areas triggering the release of various inflammatory mediators that affect uterine tone and create inhospitable environment for embryos [5,20]; such molecules may have direct embryocidal effects.

It is unlikely that dinoprostone, Cervidil® inserts and/or cervical plugs had a direct detrimental effect on fertility after TCET. Mammalian embryos secrete large quantities of PgE_2_ to facilitate their entry into the uterus and PgE_2_ also has a significant involvement in embryo implantation [21]. It was shown that estradiol upregulates PgE_2_ production by the endometrium in sheep and inhibition of PgE_2_ synthesis prevents implantation, which can be restored by the administration of endogenous PgE_2_[22].

## Conclusions

In closing, the impenetrability of the ovine cervix using conventional instruments is a major factor precluding the widespread use of TCET in sheep. Pharmacological dilation of the cervix can significantly improve the ease with which the ovine cervix can be penetrated. Cervidil®, a dinoprostone-containing vaginal insert with a slow release mechanism currently used to induce labor in women, can improve cervical penetration rates for the novel method of TCET.

## Competing interests

The authors declare that they have no competing interests.

## Authors’ contributions

Both authors contributed equally to the present work, read and approved the final manuscript.
